# Representation of women authorship in the top-cited articles published in the medical imaging literature

**DOI:** 10.1186/s13244-025-02085-4

**Published:** 2025-09-27

**Authors:** Hyung Jin Lee, Dae Young Yoon, Sora Baek, Kyoung Ja Lim, Young Lan Seo, Eun Joo Yun

**Affiliations:** 1 Department of Radiology, Seogwipo Medical Center, Seogwipo-si, Republic of Korea; 2 Department of Radiology, Kangdong Seong-Sim Hospital, Hallym University College of Medicine, Seoul, Republic of Korea

**Keywords:** Women authorship, Top-cited, Publications, Imaging literature

## Abstract

**Objective:**

To investigate the representation of women among the authors of top-cited articles published in the medical imaging literature.

**Materials and methods:**

This retrospective bibliometric study queried the Web of Science database to identify the top-cited articles (citation number ≥ 300) in the medical imaging literature. The gender of the first and last (senior) authors was determined based on online databases. The year of publication, country of origin, document type, and subspecialty for each article were also collected. We analyzed the proportion of women authors and the relationships between author gender and article characteristics.

**Results:**

Among 596 top-cited articles, women accounted for 132 (22.1%) of first authors and 84 (14.1%) of last authors. Women as last authors were more likely to publish with women first authors compared to male first authors (odds ratio: 1.35). Women's first authorship was significantly more frequent in articles from South Korea (44.4%; phi = 0.095) and in radiation oncology (38.1%; phi = 0.106) and significantly less frequent in articles from France (0.0%; phi = −0.102). Women's last authorship was significantly more frequent in articles from the Netherlands (30.6%; phi = 0.120), in breast (38.9%; phi = 0.126), and in radiation oncology (28.6%; phi = 0.115), and significantly less frequent in nuclear medicine (4.3%; phi = −0.083).

**Conclusion:**

Women authors remain underrepresented in top-cited articles published in the medical imaging literature, with country of origin and subspecialty identified as factors of influence.

**Critical relevance statement:**

Women are still underrepresented among the authors of the top-cited articles in the medical imaging literature. The findings highlight the gender disparities in the highest academic achievement in this biomedical field and provide valuable insight into this ongoing issue.

**Key Points:**

- Women authors remain underrepresented in top-cited articles in the medical imaging literature.
- Women accounted for 22.1% of first authors and 14.1% of last authors.
- There were variations in the proportion of women authors between countries and subspecialties.

**Graphical Abstract:**

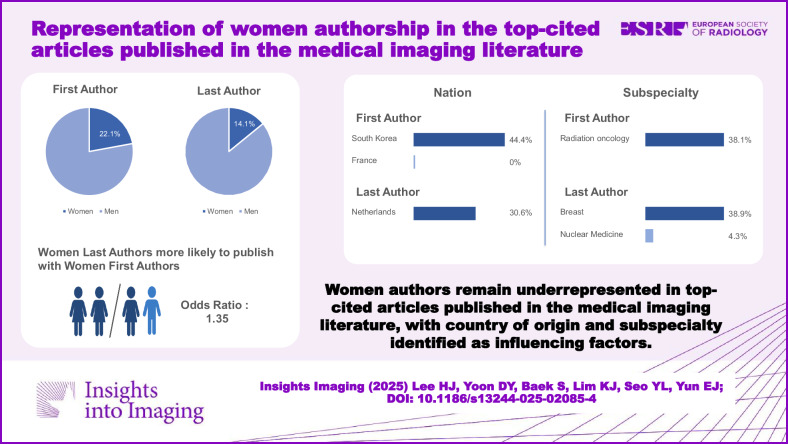

## Introduction

Nowadays, women make up more than half of medical students and physicians in many countries [1, 2]. As the proportion of women physicians continues to increase, the representation of women in medicine has progressively increased in recent decades [3]. Despite this growing trend, women remain underrepresented in all levels of rank within academic radiology, especially among senior faculty [2].

Research productivity and authorship position are strongly linked to career prospects in academic medicine [4]. Therefore, these markers are widely used as indicators of gender equity. In addition, the number of citations an article receives after its publication is commonly used as a metric for academic recognition, influence, and acceptance by the scientific community, as well as in professional evaluations and the promotion of individual faculty members [5, 6]. Furthermore, top-cited articles represent the most influential and impactful contributions to a particular field or discipline [7].

**Fig. 1:**
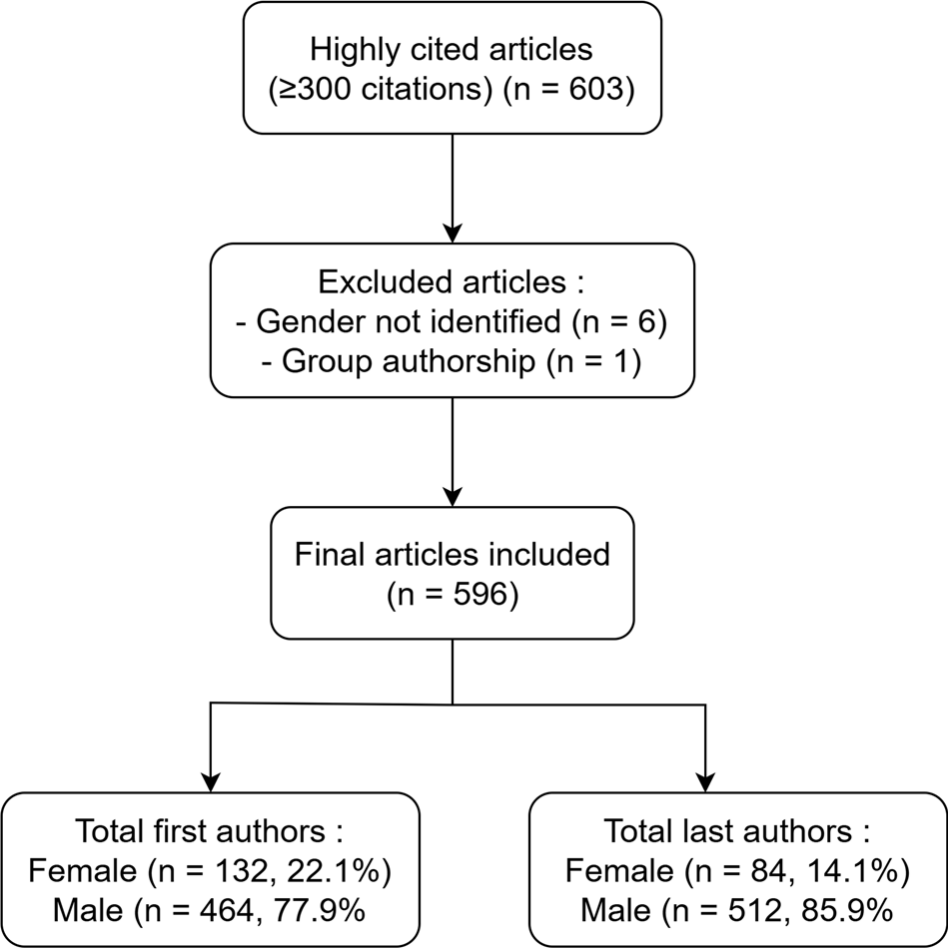
The process of selection of the highly cited articles and the total numbers of first and last authors

Many studies [8–18] have demonstrated that the scientific achievements of women in the fields of radiology and medical imaging remain underrepresented. To the best of our knowledge, no prior studies have specifically examined gender authorship in top-cited articles in this field, although studies on gender representation in medical imaging literature exist. This study addresses this gap by investigating women's author representation within the top-cited articles published in the medical imaging literature (Fig. 1).

## Materials and methods

This study was a retrospective bibliometric analysis that used only publicly available data and was thus exempt from the need for institutional review board approval.

### Inclusion and exclusion criteria

To evaluate the number of citations to articles published in the medical imaging literature, articles from 208 journals indexed in the Web of Science (Clarivate Analytics) category for the year 2023, “Radiology, Nuclear Medicine and Medical imaging” were included. The number of citations for each article was determined using the “cited reference search” tool on Web of Science, selecting the top-cited articles on September 12, 2024. All articles with ≥ 300 citations were collected and compiled into a single database in descending order based on the citation number.

Data from a total of 603 articles were collected. Six articles (1.0%) were excluded for which the gender of the first (*n* = 4) or last (senior) (*n* = 2) author could not be determined. Articles with group authorship, such as an association, corporation, or research consortium, were also excluded. Ultimately, 596 articles were included in the analysis.

### Variables extracted

After identifying top-cited articles (≥ 300 citations), the following information was extracted from each article: names and affiliated institutions of the first (i.e., first position in the authorship line) and last (i.e., last position in the authorship line) authors, gender of the first and last authors, year of publication (2013–2014, 2015–2016, or 2017–), country of origin, document type (original article, review, guideline, meta-analysis, or case report), and subspecialty [abdominal, artificial intelligence, basic science, breast, cardiac, chest, genitourinary, musculoskeletal, neuroradiology/head and neck, nuclear medicine, pediatric, radiation oncology, vascular/interventional, or miscellaneous (not conforming to one of the preceding categories)].

In cases where only the initials of the first names were used, an Internet search was conducted using search engines (Google), other publications on databases (PubMed), and institutional websites to determine the authors’ first names. The publication years were grouped into three intervals (2013–2014, 2015–2016, and 2017–present) to ensure balanced sample sizes across periods. Articles published after 2018 represented a relatively small proportion of the dataset, thus, grouping them together with 2017–2018 articles allowed for meaningful statistical comparison and analysis. The country of origin for each article was determined based on the institution of the first author. If the first author was affiliated with institutions in more than one country, the country of origin was determined on the basis of the first listed affiliation. In articles published by a single author, this author was considered both the first and last author.

Three authors (H.J.L., D.Y.Y., and S.B.) shared in extracting data from 596 top-cited articles. Questionable cases (*n* = 25, 4.2%) were decided by consensus of all three investigators.

### Identification of authors’ gender

For each top-cited article, the gender of the first and last authors was determined since these authors are traditionally considered the most important and are commonly associated with academic promotion. Gender was determined by Genderize.io (https://genderize.io), a validated gender identification software. This website analyzes first names to determine the likelihood of their being associated with a specific gender. Gender was assigned to an author if Genderize.io predicted the gender at a probability ≥ 90%.

For 123 (20.4%) of the first author names and 91 (15.1%) of the last author names that could not be classified or for which the probability of gender classification < 90%, attempts were made to gain additional information (curricula vitae or photographs) regarding the authors. Internet searches were conducted by visiting research websites (ResearchGate, LinkedIn, Academia, Google Scholar, Mendeley, etc.), institution websites, and social media platforms to identify the gender of the authors. If the gender of the first or last author could not be determined even by these additional means, then the article was excluded from analysis.

### Statistical analysis

This study adopted a descriptive research approach by means of bibliometric analysis. The primary outcomes were the gender distributions of the first and last authors of scientific articles published in the medical imaging literature. We also examined the correlation between the gender of the first and last authors. Articles with a single author were excluded from the correlation analysis between the gender of the first and last authors. A multinomial logistic regression model was used to assess whether specific first-last author gender combinations occurred at different frequencies. The dependent variable categorized author pairs into four groups: female first author-female last author, female first author-male last author, male first author-female last author, and male first author-male last author (reference group). Independent variables included publication year, country, and subspecialty. In addition, we also compared the proportions of the first and last women authors for each journal, during each study period, by document type, by country of origin, and by subspecialty, using the Chi-square test or Fisher’s exact test if appropriate.

All statistical analyses were performed using SPSS statistical software (version 21.0; IBM). *p* < 0.05 was considered statistically significant.

## Results

Among 596 top-cited articles published in the medical imaging literature, women accounted for 22.1% (132 articles) of all first authors and 14.1% (84 articles) of all last authors. The proportion of women last authors was significantly lower than the proportion of women first authors (*p* < 0.0005). Articles with women as last authors were more likely to have women first authors than those with men as last authors (odds ratio: 1.35; 95% CI: 0.80–2.30; *p* = 0.268) (Table 1).

**Table 1:** The gender of the first and last authors and their association between gender and authorship roles in the top-cited (citation number ≥ 300) articles published in the imaging literature

Gender of the first and last authors (*n* = 596)	Number
Articles published with women first author	132 (22.1%)
Articles published with men first author	464 (77.9%)
Articles published with women last author	84 (14.1%)
Articles published with men last author	512 (85.9%)

Correlation of gender of the first and last authors^a^ (*n* = 586^b^)	
Articles published with women first and women last author	22 (3.8%)
Articles published with men first and women last author	60 (10.2%)
Articles published with women first and men last author	108 (18.4%)
Articles published with the men first and the men last author	396 (67.6%)

^a^ The odds ratio of women first author in articles with women last author compared with articles with men last author was 1.35 (95% CI: 0.80–2.30; *p* = 0.268)

^b^ Ten articles with a single author (there were 8 men and 2 women) were excluded from the analysis of the correlation between the gender of the first and last authors

When ten countries with a publication share of at least 3% (*n* = 18) were analyzed, the proportion of women authors varied between countries. The proportion of women as first authors was significantly higher in articles from South Korea (44.4%; phi = 0.095; *p* < 0.05) and significantly lower in articles from France (0.0%; phi = –0.102; *p* < 0.01). Additionally, the proportion of women as last authors was significantly higher in the Netherlands (30.6%; phi = 0.120; *p* < 0.005). In contrast, no significant differences were observed in the proportions of women first and last authors of articles from the top two countries―the United States and the United Kingdom―which accounted for 34.9% and 10.6% of total publications, respectively. Proportions of women first and last authors were highest in 2013-2014 (24.9%) and 2017-present (16.4%), respectively, but these differences did not reach statistical significance. Regarding the document type, no significant difference was observed in the proportion of both women first and last authors (Table 2).

**Table 2:** The proportion of women first and last authorship of top-cited (citation number ≥ 300) articles published in the imaging literature, according to publication period, country of origin, journal, and document type

Authorship	Women (%)	Phi-coefficient^a^	*p*-value
First author			
Publication period			
2013–2014	46 (24.9%)	0.044	0.284
2015–2016	31 (20.0%)	−0.031	0.454
2017–	55 (21.5%)	−0.014	0.735
Country of origin^b^			
United States	46 (22.1%)	−0.001	0.989
United Kingdom	19 (30.2%)	0.066	0.105
Germany	7 (13.5%)	−0.065	0.114
China	13 (28.9%)	0.046	0.257
The Netherlands	6 (16.7%)	−0.033	0.414
Canada	1 (41.2%)	−0.083	0.062
France^c^	0 (0.0%)	−0.102	0.007
South Korea^c^	8 (44.4%)	0.095	0.038
Switzerlands	7 (41.2%)	0.079	0.072
Italy	4 (25.0%)	0.011	0.763
Document type			
Original article	95 (21.3%)	−0.035	0.390
Review	24 (26.1%)	0.041	0.322
Guideline	8 (21.1%)	−0.007	0.867
Meta-analysis	4 (25.0%)	0.011	0.763
Case report	1 (25.0%)	0.006	1.000
Last author			
Publication period			
2013–2014	26 (14.1%)	−0.001	0.985
2015–2016	16 (10.3%)	−0.064	0.117
2017–	42 (16.4%)	0.058	0.159
Country of origin^b^			
United States	30 (14.4%)	0.007	0.866
United Kingdom	6 (9.5%)	−0.045	0.270
Germany	6 (11.5%)	−0.023	0.597
China	4 (8.9%)	−0.043	0.297
The Netherlands^c^	11 (30.6%)	0.120	0.003
Canada	4 (18.2%)	0.023	0.534
France	0 (0.0%)	−0.077	0.058
South Korea	4 (22.2%)	0.041	0.302
Switzerlands	5 (29.4%)	0.075	0.077
Italy	0 (0.0%)	−0.067	0.146
Document type			
Original article	58 (13.0%)	−0.054	0.187
Review	12 (13.0%)	−0.013	0.753
Guideline	8 (21.1%)	0.052	0.203
Meta-analysis	4 (25.0%)	0.052	0.262
Case report	2 (50.0%)	0.085	0.097

^a^ The phi-coefficient measures and tests the strength of association between women's authorship and article variables

^b^ Statistical analysis was performed in countries with more than 3% of total publications

^c^ Statistically significant

Gender representation also varied across subspecialties. Women were statistically significantly more represented as first authors in radiation oncology (38.1%; phi = 0.106; *p* = 0.01) and last authors in breast imaging (38.9%; phi = 0.126; *p* = 0.007) and radiation oncology (28.6%; phi = 0.115; *p* = 0.005). In contrast, nuclear medicine showed a significantly lower representation of women as the last authors (4.3%; phi = −0.083; *p* < 0.05) (Table 3).

**Table 3:** The proportion of women first and last authorship of top-cited (citation number ≥ 300) articles published in the imaging literature, according to subspecialty

Authorship	Subspecialty	Women (%)	Phi-coefficient^a^	*p*-value
First author	Abdominal	6 (23.1%)	0.005	0.907
	Artificial intelligence	9 (13.0%)	−0.079	0.053
	Basic science	8 (23.5%)	0.008	0.842
	Breast	6 (33.3%)	0.048	0.253
	Cardiac	14 (27.5%)	0.039	0.340
	Chest	9 (18.0%)	−0.030	0.461
	Genitourinary	2 (14.3%)	−0.029	0.745
	Musculoskeletal	1 (25.0%)	0.006	1.000
	Neuroradiology/head/neck	34 (20.0%)	−0.033	0.425
	Nuclear medicine	12 (25.5%)	0.024	0.560
	Pediatric	2 (66.7%)	0.076	0.125
	Radiation oncology^b^	16 (38.1%)	0.106	0.010
	Vascular/interventional	0 (0.0%)	−0.062	0.210
	Miscellaneous	13 (21.7%)	−0.004	0.925
Last author	Abdominal	3 (11.5%)	−0.016	1.000
	Artificial intelligence	13 (18.8%)	0.049	0.228
	Basic science	2 (5.9%)	−0.058	0.207
	Breast^b^	7 (38.9%)	0.126	0.007
	Cardiac	3 (5.9%)	−0.072	0.078
	Chest	7 (14.0%)	−0.001	0.984
	Genitourinary	3 (21.4%)	0.033	0.431
	Musculoskeletal	0 (0.0%)	−0.033	1.000
	Neuroradiology/head/neck	23 (13.5%)	−0.010	0.802
	Nuclear medicine^b^	2 (4.3%)	−0.083	0.043
	Pediatric	0 (0.0%)	−0.029	1.000
	Radiation oncology^b^	12 (28.6%)	0.115	0.005
	Vascular/interventional	1 (12.5%)	−0.005	1.000
	Miscellaneous	8 (13.3%)	−0.007	0.858

^a^ The phi-coefficient measures and tests the strength of association between women's authorship and article variables

^b^ Statistically significant

Table 4 summarizes the 14 most prolific women authors who contributed as the first or last author of two or more top-cited articles. This list is led by Ivana Isgum, who was the last author of three articles. The most cited article by a woman first author was “Medical hyperspectral imaging: a review” by Lu Guolan, published in 2014, with 1416 citations (30th position overall). The most cited article by a woman last author was “A survey on deep learning in medical image analysis” by Clara I. Sánchez, published in 2017, which received 6887 citations (2nd position overall).

**Table 4:** Most prolific women authors who contributed as the first or last author two or more of the top-cited (citation number ≥ 300) articles published in the imaging literature

Author	No. of articles	Position on author list (no. of articles)
Isgum, Ivana	3	Last (3)
Giger, Maryellen L.	2	Last (2)
Goh, Vicky	2	Last (2)
Kuehn, Andrea A.	2	Last (2)
Luna, Beatriz	2	Last (2)
Moy, Linda	2	Last (2)
Vozenin, Marie-Catherine	2	Last (2)
Dou, Qi	2	First (2)
Gil, Maria M.	2	First (2)
Griffanti, Ludovica	2	First (2)
Leonardi, Nora	2	First (2)
Ng, Francesca	2	First (2)
Puntmann, Valentina O.	2	First (2)
Schulz-Menger, Jeanette	2	First (2)

## Discussion

Women have long been underrepresented in the authorship of scientific publications compared to men. The gender disparity in scientific publication is an important issue, reflecting missed opportunities for career advancement among women researchers.

Previous studies have investigated gender disparity across medical disciplines, reporting a wide range of proportions representing women's authorship in scientific publications. The proportions of women first or last authors reported vary greatly between disciplines, countries, impacts of journals, and publication periods [19–24]. These results could be attributed to many discipline- or country-specific differences. For example, the Association of American Medical Colleges found that 48% of residents and 45% of full-time faculty were women in the United States in 2023. In the field of radiology, however, the proportion of women residents and full-time faculty in the United States has been stable between 26% and 27% and 28% and 30% over the last decade, respectively. In addition, women were found to account for 30% of full-time faculty, 24% of professors, and 21% of department chairs in radiology departments affiliated with medical schools in the United States in 2023 [2]. Considering the dominance of the United States in top-cited articles, the underrepresentation of women found in this study could be related in part to the relatively few women in the field of radiology in the United States. In particular, the relatively lower proportion of women last authors could potentially be explained by the smaller proportion of women senior faculty members who are eligible to serve as last authors. Conventionally, the first author position indicates active involvement in the project for an early-career faculty member or trainee, while the last author is typically the principal investigator of the grant or head of the research team who is more likely to hold a higher academic rank.

This study is the first to investigate women's authorship in top-cited articles published in the medical imaging literature to date. The results indicate that women are greatly underrepresented in the primary authorship of top-cited articles. The proportions of women first (22.1%) and last (14.1%) authors as ascribed in our study are substantially lower than those reported previously in the field of radiology or medical imaging (Table 5) [8–18].

**Table 5:** Summary of studies reporting the proportion of women first, corresponding, or last authorship of articles published in radiology or medical imaging literature

Reference number	Journals	Study period (year)	Proportion of women authors (%)			Comment
			First	Corresponding	Last	
[8]	*AJR, Radiology*	1991–1993 2001–2003 2011–2013	20.4 25.8 34.4	18.0 20.3 28.7		Original research articles
[9]	*JVIR, CVIR*	2006–2017	16.0		8.7	
[10]	*AJR, AR, Radiology*	2011–2015	30.2		24.5	
[11]	*AJR, EJR, JCAT, Radiology*	1984 1994 2004 2014	12.9 21.0 25.3 31.6		9.3 16.0 13.5 21.9	Articles from American medical institutions
[12]	*ER* *CVIR*	2002–2016	27.9 14.6		16.1 5.2	
[13]	Nine high-impact American radiology journals	2002 2017	26.9 37.4		15.7 23.9	
[14]	Ten high- (4), medium- (3), low- (3) impact radiology journals	2007–2008 2017–2018	20.6 29.1		10.7 16.4	
[15]	50 high-impact radiology journals	2018–2019 2020	31.6 32.3		19.3 20.7	
[16]	*PR*	2017 2018 2019 2020	38.6 39.0 44.4 13.3	38.8 39.5 39.1 40.8	38.0 30.7 39.2 33.7	
[17]	*Radiology, AJR, JACR, AR*	2007–2020	30.3		23.3	
[18]^a^	All medical imaging literature	2018–2019 2020–2021	36.4 38.1		27.2 28.1	Web of Science category “Radiology, Nuclear Medicine and Imaging”

*AJR* American Journal of Roentgenology, *JVIR* Journal of Vascular and Interventional Radiology, *CVIR* Cardiovascular and Interventional Radiology, *AR* Academic Radiology, *EJR* European Journal of Radiology, *JCAT* Journal of Computer-Assisted Tomography, *ER* European Radiology, *PR* Pediatric Radiology, *JACR* Journal of the American College of Radiology

^a^ Corrected proportions of women authorship after excluding the articles in which the gender of authors could not be determined

The reasons for this finding are likely multifaceted. First, on average, articles by women major authors are cited less often in comparison to their male counterparts [11, 17, 25]. The cause of this finding remains unclear, but one plausible explanation proposed in previous literature is unconscious gender bias in citation practices [25]. Further research into understanding these barriers to citation for women researchers is needed to identify effective interventions. Second, different subspecialties have different sizes of scientific communities and thus different citation frequencies [26]. It is possible that women researchers are more represented in smaller subspecialties, such as breast and pediatric imaging, which generally receive fewer citations [10, 12, 14]. Third, chronological bias is present in the current analysis of top-cited articles because older articles have had more time to accumulate citations. In our study, more than half of the top-cited articles were published in 2017 or earlier. Therefore, our results could not reflect the current trend of increasing women's authorship in the field of medical imaging. Finally, the lower proportion of women's authorship observed in medical imaging may be attributable to the inclusion of subspecialties outside of radiology in our dataset, such as “miscellaneous,” “artificial intelligence,” and “basic science.” However, in a recent study by Jabal et al [18], gender disparities were evaluated across the medical imaging literature under the Web of Science category “Radiology, Nuclear Medicine and Imaging,” the same inclusion criteria used in the present study, from 2018 to 2021. Their results on the proportion of women authorship were similar to those reported for radiology journals (Table 5).

In the present study, discrepancies were also observed in the proportions of women's authorship among various countries and subspecialties, in line with previous studies in the field of radiology [8, 11, 15]. South Korea and the Netherlands showed significantly higher proportions of women's authorship compared with the other countries analyzed. In addition, radiation oncology and breast imaging were significantly dominant subspecialties in terms of women authorship (Tables 2 and 3). These results may simply be related to discrepancies in the gender distribution of physicians within the different countries or different subspecialties. Further information on the proportion of women physicians in the various countries or subspecialties would be needed to verify this finding.

Furthermore, these findings could be the result of gender-based systemic barriers for women physicians in academic medicine in general [27, 28], as well as in the medical imaging field [29, 30], including institutional (e.g., promotion inequity, small research sections, and lack of funding/resources) and social (e.g., societal norms and family responsibilities discrimination) barriers. Women may also be hindered by implicit bias in the academic medical publishing process, such as bias in peer review and invited authorship [31, 32]. This reveals another potential source of gender bias affecting the number of citations among women in highly impactful articles in the field of medical imaging. Research impact, measured by the total number of citations, *h*-index, and top-cited articles, is a key factor for achieving the highest levels of academic ranks and executive leadership positions [10, 33, 34].

This study has several limitations. First, the gender of authors was primarily determined using Genderize.io, a validated name-based gender identification software [8–18]. For names that could not be confidently classified (probability < 90%) or were ambiguous, additional manual verification was performed using institutional websites and academic profiles. In rare cases where gender could not be determined even after manual verification, the associated article was excluded from the study. However, because this concerned only a small number of authors (1.0%), this limitation likely did not affect our overall results. Secondly, only the genders of the first and last authors were determined based on the assumption that these authorship positions are the most important in research activities and publications. As a result, other authorship positions, such as middle author and corresponding author, were not captured within this analysis. However, the importance of the first and last authorships with regard to academic promotions and leadership positions is established. In fact, this first-last-author-emphasis model has been widely used in previous studies on women's authorship in the literature [9–15, 17, 18].

Taken together, these results highlight the fact that despite remarkable advances in women's authorship in recent decades, women authors continue to be underrepresented among top-cited articles published in the medical imaging literature, with a notable effect regarding subspecialty and country of origin.

## Data Availability

Data generated or analyzed during the study are available from the corresponding author upon request.

## Abbreviation

CI: Confidence interval

## References

[1] OECD (2023) iLibrary. Doctors (by age, sex and category). https://www.oecd-ilibrary.org/social-issues-migration-health/health-at-a-glance-2023. Accessed 28 Nov 2024

[2] AAMC (2024) Association of American Medical Colleges website. Available via AAMC.ORG. https://www.aamc.org/data-reports/faculty-institutions/report/state-women-academic-medicine. Accessed 28 Nov 2024

[3] Jagsi R, Guancial EA, Worobey CC et al (2006) The “gender gap” in authorship of academic medical literature—a 35-year perspective. N Engl J Med 355:281–287 16855268 10.1056/NEJMsa053910

[4] Kumar N, Gupta R, Gupta S (2022) Research and academic output evaluation for career initiation or progression: critical issues for health professionals. Med Teach 44:1179–1181 35868010 10.1080/0142159X.2022.2102471

[5] Moed HF (2009) New developments in the use of citation analysis in research evaluation. Arch Immunol Ther Exp (Warsz) 57:13–18 19219533 10.1007/s00005-009-0001-5

[6] Eyre-Walker A, Stoletzki N (2013) The assessment of science: the relative merits of post-publication review, the impact factor, and the number of citations. PLoS Biol 11:e1001675 24115908 10.1371/journal.pbio.1001675 PMC3792863

[7] Garfield E (1987) 100 citation classics from the Journal of the American Medical Association. JAMA 257:52–59 3537352

[8] Yun EJ, Yoon DY, Kim B et al (2015) Closing the gender gap: increased female authorship in AJR and radiology. AJR Am J Roentgenol 205:237–241 26204270 10.2214/AJR.14.14225

[9] Xiao N, Oliveira DF, Gupta R (2018) Characterizing the impact of women in academic IR: a 12-year analysis. J Vasc Interv Radiol 29:1553–1557 30293729 10.1016/j.jvir.2018.06.010

[10] Campbell JC, Yoon SC, Grimm LJ (2019) Authorship and impact of gender-specific research in major radiology journals. J Am Coll Radiol 16:240–243 30722843 10.1016/j.jacr.2018.08.024

[11] Huang M, Naser-Tavakolian K, Clifton M et al (2019) Gender differences in article citations by authors from American institutions in major radiology journals. Cureus 11:e5313 31592368 10.7759/cureus.5313 PMC6773454

[12] Bernard C, Pommier R, Vilgrain V, Ronot M (2020) Gender gap in articles published in European Radiology and cardiovascular and Interventional Radiology: evolution between 2002 and 2016. Eur Radiol 30:1011–1019 31506817 10.1007/s00330-019-06390-7

[13] Jalilianhasanpour R, Chen H, Caffo B, Johnson P, Beheshtian E, Yousem DM (2020) Are women disadvantaged in academic radiology? Acad Radiol 27:1760–1766 33158703 10.1016/j.acra.2020.09.019

[14] Molwitz I, Yamamura J, Ozga AK et al (2021) Gender trends in authorships and publication impact in Academic Radiology—a 10-year perspective. Eur Radiol 31:8887–8896 34009412 10.1007/s00330-021-07928-4 PMC8589752

[15] Quak E, Girault G, Thenint MA, Weyts K, Lequesne J, Lasnon C (2021) Author gender inequality in medical imaging journals and the COVID-19 pandemic. Radiology 300:E301–E307 33724061 10.1148/radiol.2021204417 PMC7983071

[16] Ayyala RS, Trout AT (2022) Gender trends in authorship of Pediatric Radiology publications and impact of the COVID-19 pandemic. Pediatr Radiol 52:868–873 34671821 10.1007/s00247-021-05213-6 PMC8528561

[17] Xiao N, Marquez-Karry R, Oliveira DF, Berggruen S, Horowitz JM (2023) Gender disparities in academic radiology authorship: a 13-year review. Acad Radiol 30:1714–1720 36424312 10.1016/j.acra.2022.10.031

[18] Jabal MS, Ibrahim MK, McDonald JS et al (2024) The effect of the COVID-19 pandemic on academic research gender disparities in radiology. Acad Radiol 31:1265–1271 37863777 10.1016/j.acra.2023.09.032

[19] Farhan SA, Shahid I, Siddiqi J, Khosa F (2020) Assessing the gap in female authorship in neurosurgery literature: a 20-year analysis of sex trends in authorship. World Neurosurg 141:e661–e669 32522642 10.1016/j.wneu.2020.05.248

[20] Jabbarpour Y, Wilkinson E, Coffman M, Mieses A (2020) Has female authorship in family medicine research evolved over time? Ann Fam Med 18:496–502 33168677 10.1370/afm.2584 PMC7708287

[21] Zillioux J, Tuong M, Patel N, Shah J, Rapp DE (2021) Trends in female authorship within urologic literature: a comparison of 2012 and 2017. Urology 150:35–40 32890625 10.1016/j.urology.2020.08.039

[22] Hart KL, Boitano LT, Tanious A et al (2022) Trends in female authorship in high impact surgical journals between 2008 and 2018. Ann Surg 275:e115–e123 32590539 10.1097/SLA.0000000000004057

[23] Ghattas YS, Kyin C, Grise A (2023) Trends in female authorship in orthopaedic literature from 2002 to 2021: an analysis of 168,451 authors. J Bone Joint Surg Am 105:1285–1294 37155604 10.2106/JBJS.22.01290

[24] Yamamura J, Molwitz I, Ozga AK et al (2023) Gender differences and cooperation in medical authorships-an analysis of the recent ten years in five key medical disciplines. BMC Med Educ 23:68 36707803 10.1186/s12909-023-04041-6 PMC9883917

[25] Chatterjee P, Werner RM (2021) Gender disparity in citations in high-impact journal articles. JAMA Netw Open 4:e2114509 34213560 10.1001/jamanetworkopen.2021.14509 PMC8254129

[26] Yoon DY, Yun EJ, Ku YJ et al (2013) Citation classics in radiology journals: the 100 top-cited articles, 1945–2012. AJR Am J Roentgenol 201:471–481 23971438 10.2214/AJR.12.10489

[27] Jagsi R, Griffith KA, DeCastro RA, Ubel P (2014) Sex, role models, and specialty choices among graduates of US medical schools in 2006–2008. J Am Coll Surg 218:345–352 24468225 10.1016/j.jamcollsurg.2013.11.012

[28] Mamtani M, Shofer F, Mudan A et al (2020) Quantifying gender disparity in physician authorship among commentary articles in three high-impact medical journals: an observational study. BMJ Open 10:e034056 32102817 10.1136/bmjopen-2019-034056 PMC7044872

[29] Weigel KS, Kubik-Huch RA, Gebhard C (2020) Women in radiology: Why is the pipeline still leaking and how can we plug it? Acta Radiol 61:743–748 31648538 10.1177/0284185119881723

[30] Hamidizadeh R, Jalal S, Pindiprolu B et al (2018) Influences for gender disparity in the radiology societies in North America. AJR Am J Roentgenol 211:831–838 30063373 10.2214/AJR.18.19741

[31] Erren TC, Groß JV, Shaw DM, Selle B (2014) Representation of women as authors, reviewers, editors in chief, and editorial board members at 6 general medical journals in 2010 and 2011. JAMA Intern Med 174:633–635 24566922 10.1001/jamainternmed.2013.14760

[32] Murray D, Siler K, Lariviére V et al (2018) Gender and international diversity improves equity in peer review. Preprint at https://www.biorxiv.org/content/10.1101/400515v1

[33] Goswami AK, Kokabi N, Khaja MS et al (2022) Academic radiology in the United States: defining gender disparities in faculty leadership and academic rank. Acad Radiol 29:714–725 34176728 10.1016/j.acra.2021.05.016

[34] Kubik-Huch RA, Vilgrain V, Krestin GP et al (2020) Women in radiology: gender diversity is not a metric—it is a tool for excellence. Eur Radiol 30:1644–1652 31802213 10.1007/s00330-019-06493-1 PMC7033068

